# Supplementary material for: P-TRAP: a Panicle Trait Phenotyping tool
Source: BMC Plant Biol. 2013 Aug 29;13:122. doi: 10.1186/1471-2229-13-122 (PMC3848748; doi:10.1186/1471-2229-13-122)
Supplement: Additional file 7 — P-TRAP source code. The Netbeans project that contains the source code of the application. [file 1471-2229-13-122-S7.zip › javadoc.zip!/javadoc/org-bioalg-widgetfactory/org/bioalg/widgetfactory/package-frame.html]

org.bioalg.widgetfactory (WidgetFacory)


# org.bioalg.widgetfactory

## Classes

- CircleWidget
- ParticleWidget
- WidgetPopUpMenuHandler

## Enums

- WidgetPopUpMenuHandler.WIDGET\_COMMANDS
